# Serum Catestatin Levels and Their Association with Pain Severity in Fibromyalgia Syndrome: A Cross-Sectional Case–Control Study

**DOI:** 10.3390/jcm15187332

**Published:** 2026-09-21

**Authors:** Bilgehan Kolutek Ay, Muhammed Seyithanoğlu, Ayşegül Reyhan Kuzu, Arif Gülkesen

**Affiliations:** 1 Department of Physical Medicine and Rehabilitation, Faculty of Medicine, Kahramanmaraş Sütçü İmam University, Kahramanmaraş 46050, Türkiye; aysegulreyhan1997@gmail.com; 2 Department of Biochemistry, Faculty of Medicine, Kahramanmaraş Sütçü İmam University, Kahramanmaraş 46050, Türkiye; dr.muh.seyit@gmail.com; 3 Department of Physical Medicine and Rehabilitation, Faculty of Medicine, Fırat University, Elazığ 23119, Türkiye; agulkesen@hotmail.com

**Keywords:** biomarkers, catestatin, chronic pain, fibromyalgia, quality of life

## Abstract

**Background:** Catestatin (CST) is a neuropeptide involved in autonomic regulation, inflammation, and immune responses. This study aimed to compare serum CST levels between patients with fibromyalgia syndrome (FMS) and healthy controls and to examine their associations with pain severity, disease impact, and fibromyalgia-specific quality of life. **Methods:** Forty women with FMS and 40 healthy women were included. Serum CST levels were measured by enzyme-linked immunosorbent assay. Pain severity, disease impact, and quality of life were assessed using the Visual Analog Scale (VAS), Fibromyalgia Impact Questionnaire (FIQ), and Fibromyalgia Quality of Life Scale (FM-QoLS), respectively. **Results:** No statistically significant difference in serum CST levels was detected between women with FMS and healthy women in this sample (*p* = 0.111). CST was inversely correlated with daytime (ρ = −0.510, *p* = 0.001) and nighttime pain (ρ = −0.364, *p* = 0.021); after correction for multiple comparisons, only the association with daytime pain remained significant. In multivariable analyses, lower CST levels remained associated with greater daytime pain severity after adjustment for age, Body Mass Index, and symptom duration; an inverse association was also observed for nighttime pain, although findings for nighttime pain were less consistent across analyses. CST was not significantly associated with symptom duration, FIQ, or FM-QoLS scores. **Conclusions:** Serum CST levels did not differ significantly between women with FMS and healthy women in this sample. Within the FMS group, lower CST levels were associated with greater pain severity, with the most consistent association observed for daytime pain. These findings should be considered exploratory and hypothesis-generating and do not establish CST as a diagnostic, prognostic, or clinically useful biomarker for FMS. Further studies in larger and more diverse populations are needed to confirm these findings and determine their clinical relevance.

## 1. Introduction

Fibromyalgia syndrome (FMS) is a heterogeneous chronic pain disorder characterized by widespread musculoskeletal pain, fatigue, sleep disturbances, cognitive symptoms, and a marked reduction in quality of life. FMS is more frequently identified in women, and sex-related differences in pain sensitivity and clinical presentation have been reported [1,2]. Although central sensitization is considered one of the key mechanisms underlying the pathophysiology of FMS, current evidence suggests that the disease cannot be explained solely by alterations in pain processing pathways. Autonomic nervous system dysfunction, neuroimmune interactions, neuroendocrine alterations, and low-grade systemic inflammation have also been implicated in the development and persistence of FMS symptoms [2,3,4]. In recent years, various biomarkers and biological pathways have been investigated to improve the understanding of the mechanisms underlying fibromyalgia. However, no specific and reliable biomarker suitable for clinical use has yet been identified. Therefore, current research has shifted its focus from the search for a single biomarker toward a better understanding of the neurobiological and immunological mechanisms underlying the disease [3,4,5,6].

Catestatin (CST) is a biologically active neuroendocrine peptide derived from chromogranin A and was first described by Mahata et al. [7]. CST has been shown to inhibit catecholamine release, regulate autonomic nervous system activity, and exert modulatory effects on inflammation and immune responses [8,9]. Alterations in circulating CST levels have also been reported in hypertension, further supporting its involvement in cardiovascular and autonomic regulation [10,11]. In recent years, increasing attention has been directed toward the potential role of CST in inflammatory, rheumatic, and chronic pain conditions. Although alterations in serum CST levels have been reported in several rheumatic diseases and CST has been associated with various clinical disease characteristics, the available findings appear to vary across different disease populations [12,13]. These observations suggest that the role of CST may be closely related to disease-specific pathophysiological mechanisms. An overview of previous studies investigating CST in rheumatic and pain-related conditions is provided in Table 1.

In addition to pain and other symptoms, impaired quality of life represents one of the most important clinical outcomes in patients with FMS. Therefore, assessment of quality of life constitutes an important component of disease evaluation. The Fibromyalgia Quality of Life Scale (FM-QoLS), which was recently developed and validated in the Turkish population, is a disease-specific instrument designed to assess quality of life in patients with FMS [16]. However, data regarding its use in clinical research remain limited.

In the current literature, no previous study has evaluated serum CST levels in patients with FMS. Therefore, the aim of the present study was to compare serum CST levels between women with FMS and healthy women and to investigate the association between CST levels, pain severity, disease impact, and FMS-specific quality of life.

## 2. Methods

### 2.1. Study Design

This cross-sectional case–control study was designed and reported in accordance with the Strengthening the Reporting of Observational Studies in Epidemiology (STROBE) guidelines. The aim of the study was to evaluate serum CST levels in patients with FMS and to investigate the relationships between CST levels, pain severity, disease impact, and quality of life.

### 2.2. Sample Size

The initial sample size was estimated a priori using G*Power version 3.1.9.7. Because no FMS-specific data on serum CST levels were available at the time of study planning, the effect-size estimate was derived from the rheumatoid arthritis study by Simac et al. [13], which evaluated the same circulating biomarker in a rheumatic disease population and provided the between-group serum CST data required to estimate a standardized effect size. In that study, the final mean serum CST levels were 10.53 ± 3.90 ng/mL in patients with rheumatoid arthritis and 5.24 ± 2.37 ng/mL in healthy controls. Based on Cohen’s d = 1.639, using a two-sided independent-samples *t*-test with α = 0.05, 95% power, and equal allocation between groups, the a priori calculation yielded a total sample requirement of 22 participants (11 in each group). Nevertheless, 80 participants (40 patients with FMS and 40 healthy controls) were included. Given the uncertainty associated with extrapolating the effect size from a different disease population, a sensitivity analysis based on the achieved sample size was also performed. For the final sample of 40 participants in each group, a two-sided α = 0.05 corresponded to 80% power to detect a standardized between-group difference of d ≥ 0.634. This sensitivity analysis was intended to characterize the detectable between-group effect with the achieved sample size and should not be interpreted as demonstrating adequate power for all secondary or exploratory analyses.

### 2.3. Participants

The FMS group consisted of 40 consecutive female patients who attended the outpatient clinic of the Department of Physical Medicine and Rehabilitation, Kahramanmaraş Sütçü İmam University Faculty of Medicine, between 8 December 2025 and 19 January 2026. FMS was newly diagnosed according to the 2016 revised American College of Rheumatology (ACR) criteria [17], and patients who met the eligibility criteria were included in the study. As part of the diagnostic evaluation, the Widespread Pain Index (WPI) and Symptom Severity Scale (SSS) were assessed in patients with FMS according to the 2016 revised ACR criteria. However, individual WPI and SSS scores were not retained as separate research variables and were therefore unavailable for further analyses.

The control group consisted of 40 healthy female volunteers who were recruited between 19 January and 1 March 2026. During recruitment of the control group, the age distribution of the FMS group was used as a reference, and healthy controls were recruited to obtain a comparable overall age distribution between the two groups. Matching was performed at the group level, without individual patient–control pairing.

### 2.4. Eligibility Criteria

*FMS group*: Participants aged 18–65 years were eligible for inclusion. Individuals with diabetes mellitus, morbid obesity, hypertension, heart failure, coronary artery disease, thyroid disorders, psychiatric disorders, osteoporosis, a history of malignancy, systemic inflammatory or rheumatic diseases (including rheumatoid arthritis, ankylosing spondylitis, and connective tissue diseases), clinically significant cardiac arrhythmias, acute or chronic infections, pregnancy, or breastfeeding were excluded. Current smokers were also excluded.

Current medication use was systematically assessed at enrollment. Individuals who had used medications commonly employed in the pharmacological management of FMS, including duloxetine, pregabalin, and amitriptyline, during the preceding six months were excluded, irrespective of the indication for which these medications had been prescribed (e.g., neuropathic pain, neuralgia, migraine, or radicular pain). Individuals receiving other psychiatric medications or alpha- or beta-adrenergic blockers were also excluded because of their potential influence on pain-related, neuroendocrine, or autonomic pathways relevant to the study. The use of simple analgesics and nonsteroidal anti-inflammatory drugs (NSAIDs) was also specifically assessed at enrollment, and individuals who had used these medications within the preceding seven days were excluded. No pharmacological or non-pharmacological treatment was discontinued, withheld, or delayed for the purpose of study participation. Study assessments were completed after the diagnosis of FMS had been established and before initiation of routine FMS treatment.

*Healthy control group:* Healthy female controls aged 18–65 years were recruited on a voluntary basis from the hospital and the community. A detailed medical and pain history was obtained, and available electronic health records in the national e-Nabız system were reviewed to identify any documented chronic or systemic diseases, including FMS and chronic pain conditions. Individuals with a self-reported or documented history of FMS or chronic pain, as well as those reporting regional or widespread pain, were not eligible for inclusion as healthy controls. Formal WPI and SSS assessments were not administered to the healthy controls.

The exclusion criteria regarding medical conditions, smoking, and medication use applied to the FMS group were also applied to potential controls.

### 2.5. Clinical Variables

FMS symptom duration was defined as the time from the patient-reported onset of FMS-related symptoms to the diagnostic evaluation and was determined from the clinical history obtained at enrollment.

*Pain intensity:* Pain intensity was quantified using a 10 cm Visual Analog Scale (VAS), with the left endpoint representing “no pain” and the right endpoint representing “worst imaginable pain.” Participants placed a mark on the line corresponding to their perceived pain severity, and the score was determined by measuring the distance in centimeters from the left endpoint to the participant’s mark, yielding a value from 0 to 10. Patients were asked to rate their average daytime and nighttime pain intensity during the preceding week. Daytime pain was defined as the average pain experienced during daytime hours, whereas nighttime pain was defined as the average pain experienced during nighttime hours. Pain assessments were performed during the diagnostic evaluation, before blood sampling.

*Fibromyalgia Impact Questionnaire (FIQ):* The FIQ was used to quantify the overall impact of FMS on patients’ daily lives. It covers several domains related to functional ability, well-being, work-related difficulties, and common FMS symptoms, including pain, fatigue, stiffness, anxiety, and depression. Higher total scores reflect greater disease impact and symptom burden. The validated Turkish version of the questionnaire was used [18].

**Figure 1:**
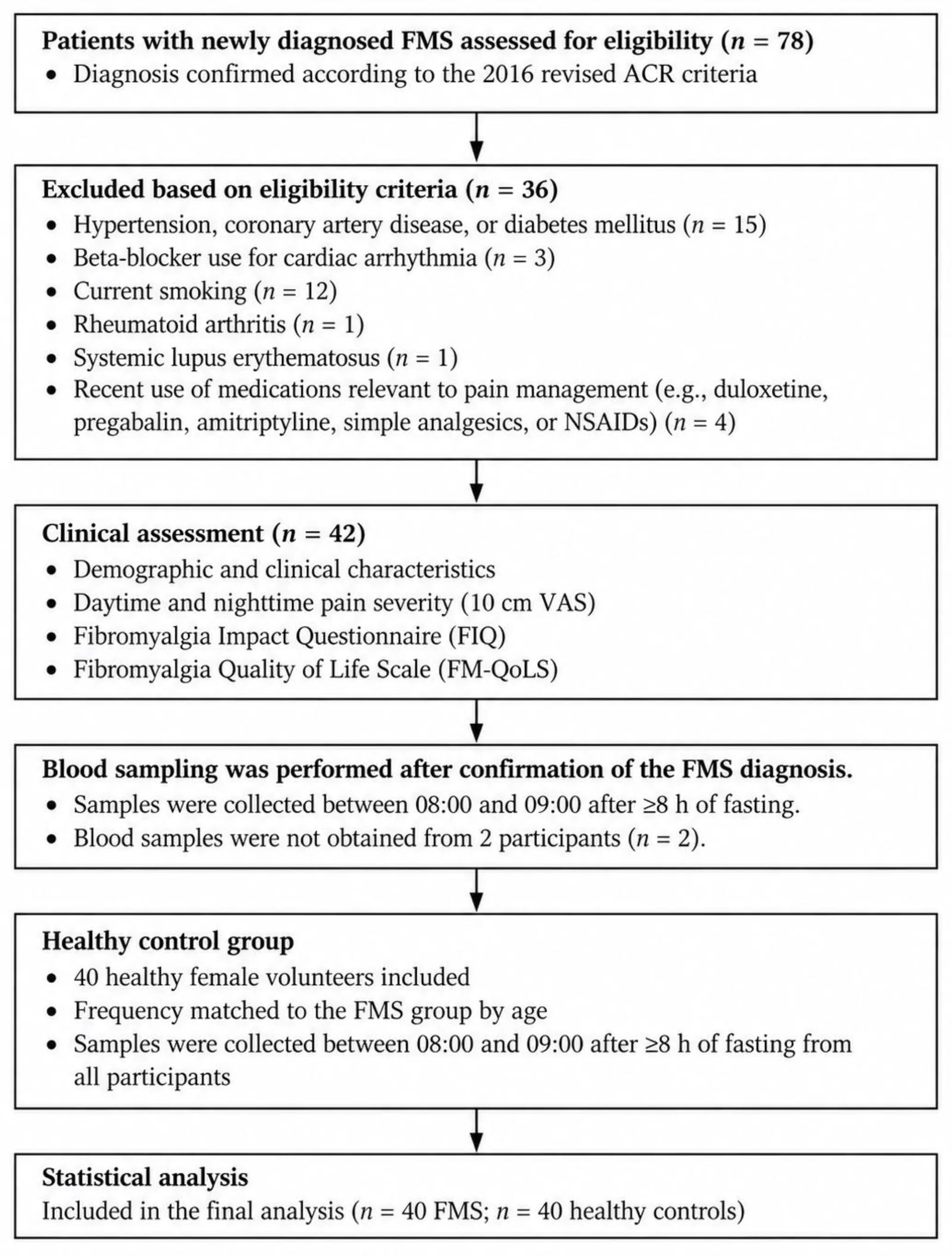
Flowchart of the study. Abbreviations: FMS, fibromyalgia syndrome; ACR, American College of Rheumatology; NSAIDs, nonsteroidal anti-inflammatory drugs; VAS, Visual Analog Scale; FIQ, Fibromyalgia Impact Questionnaire; FM-QoLS, Fibromyalgia Quality of Life Scale.

*FM-QoLS:* Fibromyalgia-specific quality of life was assessed using the FM-QoLS. The scale consists of 14 items and evaluates quality of life across two domains: symptomatology/functionality and psychosocial well-being [16]. Higher FM-QoLS scores indicate better quality of life (Figure 1).

### 2.6. Laboratory Variables and Serum CST

Venous blood samples were collected from the antecubital vein between 08:00 and 09:00 a.m. after an overnight fast of at least 8 h. In patients with FMS, blood sampling was performed only after the diagnosis of FMS had been confirmed. Patients whose diagnostic evaluation was completed later in the day returned for blood sampling the following morning or, when necessary, on the next working day. Before blood collection, participants rested in a seated position for approximately 10 min. The same standardized blood collection protocol, including the sampling time, fasting period, and pre-sampling rest, was applied to both the FMS and healthy control groups. Hematological, biochemical, lipid, and inflammatory parameters were analyzed using standard laboratory methods at the Central Laboratory of Kahramanmaraş Sütçü İmam University Faculty of Medicine. The following laboratory parameters were recorded: hemoglobin, white blood cell count, platelet count, C-reactive protein, erythrocyte sedimentation rate, creatinine, alkaline phosphatase, alanine aminotransferase, aspartate aminotransferase, total cholesterol, triglycerides, HDL-cholesterol, LDL-cholesterol, thyroid-stimulating hormone, and 25-hydroxyvitamin D. These laboratory parameters were recorded to evaluate potential metabolic, inflammatory factors that might influence serum CST levels and to ensure biological comparability between the study groups.

For serum CST analysis, blood samples were allowed to clot for 10–20 min and were subsequently centrifuged at 4000 rpm (approximately 2930× *g*) for 10 min using an NF 1200R centrifuge (Nüve, Ankara, Türrkiye). The separated serum samples were stored at −80 °C until analysis. The first blood sample was collected on 9 December 2025 and the final blood sample was collected on 1 March 2026. The maximum interval between blood collection and CST analysis was 84 days, corresponding to the first blood sample collected on 9 December 2025 and analyzed on 3 March 2026, which was within the 3-month maximum storage period specified by the manufacturer. Samples were thawed once at room temperature on the day of analysis, and repeated freeze–thaw cycles were avoided. On the day of analysis, samples were measured using a commercially available ELISA kit (E4996Hu; Lot No. 202602007; Bioassay Technology Laboratory, Shanghai, China), with a measurement range of 0.10–40 ng/mL and a detection limit of 0.046 ng/mL. The ELISA was performed according to the manufacturer’s instructions.

All 80 study samples were analyzed on 3 March 2026 in a single analytical run on the same 96-well plate using a single kit lot. Calibration standards were analyzed in duplicate, whereas study samples were analyzed as single determinations. The duplicate CVs of the calibration standards ranged from 1.21% to 5.39%, and the standard curve had an R^2^ of 0.99. These calibration-standard CVs were used to assess the consistency of the calibration measurements and were not interpreted as estimates of analytical precision for individual serum samples. Laboratory personnel performing the CST measurements were blinded to participant group allocation. Because all study samples were analyzed on a single plate in one analytical run, a study-specific inter-assay CV was not applicable. Because study samples were analyzed as single determinations, a study-specific intra-assay CV based on replicate measurements of individual samples could not be calculated. According to the manufacturer’s validation data, the intra-assay CV ranged from 3.15% to 5.31%, and the inter-assay CV was <10%. The manufacturer’s assay documentation states that the microplate is pre-coated with an antibody directed against human catestatin and that detection is performed using a biotinylated human catestatin antibody. However, quantitative specificity/cross-reactivity data for related peptides were not identified in the available manufacturer documentation.

### 2.7. Ethics Approval

Ethical approval for the study was granted by the Fırat University Non-Interventional Research Ethics Committee on 15 September 2025 (Decision No. 700788). The ethical review covered participant recruitment and sample collection at Kahramanmaraş Sütçü İmam University, where the study was conducted, and the relevant institutional authorization was obtained from the participating institution. Before any study-related procedures were undertaken, each participant provided written informed consent. All procedures were carried out in accordance with the principles outlined in the Declaration of Helsinki.

### 2.8. Statistical Analysis

Statistical analyses were performed using IBM SPSS Statistics for Windows, Version 22.0 (IBM Corp., Armonk, NY, USA). There were no missing data for the variables included in the analyses. The distribution of continuous variables was assessed using the Shapiro–Wilk test together with visual inspection of histograms, Q–Q plots, and boxplots. Normally distributed variables were summarized as mean ± standard deviation (SD), whereas non-normally distributed variables were presented as median and interquartile range (IQR).

The primary analysis was the comparison of serum CST concentrations between the FMS and healthy control groups. Analyses examining relationships between CST and clinical variables within the FMS group, including correlation analyses and multivariable models evaluating the associations between CST and pain severity, were planned a priori as secondary analyses. Given that the sample-size calculation was based on the primary between-group comparison, these secondary analyses were interpreted as exploratory and hypothesis-generating.

Between-group comparisons were performed using an independent-samples *t*-test when the distributional assumptions for parametric analysis were met; otherwise, the Mann–Whitney *U* test was used. For normally distributed variables, between-group estimates are presented as mean differences with 95% confidence intervals (CIs), together with Cohen’s *d* as the effect-size measure. For non-normally distributed variables, Hodges–Lehmann estimates with 95% CIs and rank-biserial correlations (*r*rb) are reported. The direction of these estimates was defined as FMS minus control.

Relationships among CST and demographic, clinical, and laboratory measures were examined using Spearman’s rank correlation coefficient (ρ). Multiplicity within the correlation analyses was addressed using the Benjamini–Hochberg false discovery rate (FDR) procedure. Correction was performed separately for the 15 unique pairwise clinical correlations within the FMS group and the 17 correlations of CST with demographic and laboratory variables in the overall sample. Both unadjusted *p* values and FDR-adjusted *p* values (*q* values) are reported. For these analyses, statistical significance after multiplicity correction was defined as *q* < 0.05.

For the principal correlations between serum CST and clinical parameters within the FMS group, 95% CIs for Spearman’s ρ were additionally estimated using bias-corrected and accelerated (BCa) bootstrap resampling with 5000 paired resamples.

Separate multiple linear regression models were fitted for daytime and nighttime VAS scores to evaluate the associations between serum CST and pain severity after adjustment for potential confounding variables. CST, age, BMI, and symptom duration were entered simultaneously into each model. These two regression models were treated as exploratory analyses distinct from the prespecified correlation families and were therefore not included in the FDR correction; their two-sided *p* values and 95% CIs are reported without multiplicity adjustment. Age and BMI were included as demographic and anthropometric potential confounders, respectively, while symptom duration was included to account for differences in the duration of FMS. Model assumptions were examined through residual-versus-predicted plots and normal probability plots of residuals. Tolerance and variance inflation factor (VIF) values were used to assess multicollinearity, and standardized residuals, studentized deleted residuals, leverage values, Mahalanobis distance, and Cook’s distance were examined to identify potentially influential observations. In a separate influence sensitivity analysis, both regression models were re-estimated after excluding observations with leverage values greater than 2 k/n, where k represents the number of estimated regression parameters including the intercept and n represents the sample size. With five estimated parameters and *n* = 40, the leverage threshold was 0.25; three observations exceeded this threshold and were excluded from the sensitivity analyses (*n* = 37). This analysis was used to assess the sensitivity of the CST coefficient estimates to high-leverage observations. As a separate sensitivity analysis, the regression models were re-estimated using the Huber–White sandwich covariance estimator implemented in the GENLIN procedure of IBM SPSS Statistics; no additional finite-sample correction was applied. This analysis was used to assess the robustness of standard-error estimation and statistical inference, rather than the sensitivity of the regression coefficient estimates to influential observations. Results from the conventional linear regression models are presented as unstandardized coefficients (B) with 95% CIs and standardized coefficients (β).

All statistical tests were two-sided. For analyses not subjected to FDR correction, *p* < 0.05 was considered statistically significant; for the correlation analyses subjected to FDR correction, significance was determined using *q* < 0.05.

## 3. Results

A total of 40 women with FMS and 40 healthy women were included in the study. The clinical characteristics of patients with FMS are presented in Table 2. The median symptom duration was 24.00 months (IQR, 9.75–60.00), with moderate-to-severe symptom burden reflected by VAS, FIQ, and FM-QoLS scores.

No statistically significant between-group differences were observed in the demographic or laboratory variables presented in Table 3; additionally, metabolic and routine laboratory parameters are provided in Supplementary Table S1. Serum CST levels were 2.07 (1.77–3.34) ng/mL in the FMS group and 2.36 (1.99–4.74) ng/mL in the control group. The Hodges–Lehmann location-shift estimate (FMS minus control) was −0.285 ng/mL (95% CI, −0.720 to 0.060), with a rank-biserial correlation of −0.207 (*p* = 0.111).

Among patients with FMS, serum CST levels were negatively correlated with daytime pain intensity (VAS-day; ρ = −0.510, unadjusted *p* = 0.001) and nighttime pain intensity (VAS-night; ρ = −0.364, unadjusted *p* = 0.021). After Benjamini–Hochberg FDR correction for the 15 unique pairwise correlations, the association between CST and VAS-day remained statistically significant (q = 0.008), whereas the association with VAS-night did not (q = 0.053). No significant correlations were observed between serum CST levels and symptom duration, FIQ, or FM-QoLS scores. VAS-day and VAS-night scores were positively correlated (ρ = 0.721, unadjusted *p* < 0.001), and FIQ scores were positively correlated with both VAS-day (ρ = 0.400, unadjusted *p* = 0.011) and VAS-night (ρ = 0.480, unadjusted *p* = 0.002). FIQ and FM-QoLS scores were inversely correlated (ρ = −0.485, unadjusted *p* = 0.002). These associations remained statistically significant after FDR correction (Table 4).

Bootstrap confidence intervals for the principal correlations between serum CST and clinical parameters are provided in Supplementary Table S2. The 95% BCa bootstrap CI was −0.746 to −0.164 for the CST–VAS-day correlation and −0.642 to 0.000 for the CST–VAS-night correlation, whereas the confidence intervals for the correlations with FIQ and FM-QoLS included zero.

In the overall study population, no significant correlation was observed between serum CST and CRP levels (ρ = −0.096, unadjusted *p* = 0.399, q = 0.617). A weak positive correlation was observed between serum CST and TSH levels (ρ = 0.257, unadjusted *p* = 0.021); however, this association was no longer statistically significant after Benjamini–Hochberg FDR correction (q = 0.357). No other statistically significant correlations were observed between serum CST and the evaluated demographic or laboratory parameters after FDR correction.

In multiple linear regression analyses adjusted for age, BMI, and symptom duration, the inverse associations between serum CST and both daytime and nighttime pain intensity persisted. CST was associated with VAS-day (B = −0.175, 95% CI −0.267 to −0.083; β = −0.541; *p* < 0.001) and VAS-night (B = −0.222, 95% CI −0.336 to −0.107; β = −0.530; *p* < 0.001). For nighttime pain, the standardized CST coefficient was similar in the simple regression model (β = −0.521) and the multivariable model (β = −0.530), indicating little change in the estimated linear association after adjustment for age, BMI, and FMS symptom duration. Symptom duration was also positively associated with VAS-night (B = 0.035, 95% CI 0.001–0.069; β = 0.318; *p* = 0.041). The complete regression models are presented in Table 5. Regression diagnostics did not indicate problematic multicollinearity (VIF range, 1.004–1.361), and standardized residuals remained within ±3 in both models. Although relatively high leverage values were observed for some participants, primarily related to extreme CST values, these observations were not accompanied by extreme residuals or excessive influence (maximum Cook’s distance: 0.278 for VAS-day and 0.314 for VAS-night); therefore, all participants were retained in the main regression analyses. Sensitivity analyses using the Huber–White sandwich covariance estimator yielded unchanged CST coefficient estimates, with the associations remaining statistically significant for both VAS-day (B = −0.175, robust SE = 0.028, 95% Wald CI −0.230 to −0.119; *p* < 0.001) and VAS-night (B = −0.222, robust SE = 0.036, 95% Wald CI −0.293 to −0.150; *p* < 0.001). These analyses address the robustness of standard-error estimation and statistical inference rather than the sensitivity of the coefficient estimates to influential observations.

In a separate influence sensitivity analysis excluding the three high-leverage observations (*n* = 37), the inverse CST coefficients remained statistically significant for both VAS-day (B = −0.373, 95% CI −0.658 to −0.088; β = −0.420; *p* = 0.012) and VAS-night (B = −0.394, 95% CI −0.750 to −0.037; β = −0.337; *p* = 0.031). Although the direction of the associations was preserved, the changes in coefficient magnitude indicate some sensitivity of the effect estimates to these observations.

## 4. Discussion

In the present study, serum CST levels did not differ significantly between patients with FMS and healthy controls. In patients with FMS, serum CST levels were inversely correlated with daytime pain severity, and this association remained significant after correction for multiple comparisons. Although an inverse correlation was also observed between CST levels and nighttime pain severity in the unadjusted analysis, it did not remain significant after FDR correction. In the multivariable analyses, the association between lower serum CST levels and greater daytime pain severity persisted after adjustment for age, BMI, and symptom duration No significant associations were observed between serum CST levels and symptom duration, FIQ, or FM-QoLS scores.

CST is a neuroendocrine peptide derived from chromogranin A and was initially characterized through its effects on catecholamine release and cardiovascular regulation. Its involvement in autonomic, inflammatory, and immune pathways has subsequently prompted investigation of circulating CST levels in a range of chronic diseases. More recently, CST has also been investigated in rheumatoid arthritis, ankylosing spondylitis, and pain-related conditions [7,9,12,13].

The number of studies evaluating the relationship between CST levels and rheumatic diseases remains limited. In a study conducted by Simac et al., which included 80 patients with rheumatoid arthritis and 80 healthy controls, serum CST levels were found to be significantly higher in the rheumatoid arthritis group. The same study also reported positive correlations between CST levels and symptom duration, Disease Activity Score in 28 Joints, and Health Assessment Questionnaire scores. In contrast, a study conducted in Türkiye including 95 patients with ankylosing spondylitis and 85 healthy controls reported significantly lower serum CST levels in the patient group, with CST levels being negatively associated with disease activity [12,13].

The differences observed between previous studies and our findings may reflect not only disease-specific biological differences but also variations in study populations and treatment characteristics. In the rheumatoid arthritis study, all patients were receiving disease-modifying antirheumatic drugs, biologic agents, or Janus kinase inhibitors, whereas patients in the ankylosing spondylitis study were evaluated while receiving various antirheumatic treatments, including nonsteroidal anti-inflammatory drugs, sulfasalazine, and biologic agents [12,13]. Although direct evidence regarding the effects of these treatments on serum CST levels is limited, treatment status should be considered when interpreting CST findings, as these therapies may alter inflammatory activity and cytokine-related pathways implicated in CST regulation [9]. In addition, serum CST levels have been associated with several cardiovascular, autonomic, and metabolic factors [19,20]. To reduce potential confounding, the present study applied stringent exclusion criteria, including relevant cardiovascular, metabolic, inflammatory, rheumatic, and psychiatric conditions, as well as smoking and the use of psychiatric medications and alpha- or beta-adrenergic blockers. Patients who had used medications commonly employed in the pharmacological management of FMS, including duloxetine, pregabalin, and amitriptyline, during the preceding six months were also excluded, irrespective of the indication for which these medications had been prescribed. These measures may have reduced the influence of several relevant comorbidities and treatment-related factors on serum CST levels, although residual confounding cannot be excluded.

Analytical differences should also be considered when comparing CST findings across studies. Previous studies used different commercial ELISA assays (Table 1), which may differ in their analytical characteristics and may therefore contribute to variability in measured CST concentrations. Interestingly, the ankylosing spondylitis study used the same E4996Hu assay as the present study and reported an inverse direction of association, with lower CST levels associated with greater disease activity, broadly resembling the inverse direction observed between CST and pain severity in our FMS cohort. However, the direction of CST alterations and clinical associations has not been consistent across disease populations, and similarities or differences in these findings cannot be attributed to the assay platform alone. Accordingly, although assay-related analytical differences should be considered, direct comparisons of absolute CST concentrations across studies using different assays and across different disease populations should be interpreted cautiously.

An important finding of the present study was the inverse association between serum CST levels and pain severity. The association with daytime pain remained statistically significant after FDR correction and persisted after adjustment for age, BMI, and symptom duration. In the influence sensitivity analysis, the inverse association with daytime pain also remained statistically significant after exclusion of the three high-leverage observations; however, the change in the magnitude of the CST coefficient indicates some sensitivity of the effect estimate to these observations. In contrast, the Huber–White analysis supported the robustness of statistical inference to standard-error estimation without addressing sensitivity of the coefficient estimate itself. The findings regarding nighttime pain were less consistent across analyses. Although serum CST was inversely correlated with nighttime pain, this association did not remain statistically significant after FDR correction (q = 0.053). In contrast, the inverse association persisted after adjustment for age, BMI, and symptom duration in the multivariable model and remained statistically significant after exclusion of the high-leverage observations, although the magnitude of the coefficient also changed. For nighttime pain, the similar estimates obtained in the simple and adjusted regression models suggest that adjustment for age, BMI, and FMS symptom duration had little influence on the estimated linear association with CST. However, this finding should be distinguished from the Spearman correlation analysis, in which the association with nighttime pain did not remain statistically significant after FDR correction. The regression findings should therefore be interpreted as complementary exploratory evidence rather than as confirmation of the FDR-adjusted correlation finding. According to the full-sample multivariable regression analyses, each 1 ng/mL higher serum CST concentration was associated with approximately 0.18-point and 0.22-point lower daytime and nighttime VAS scores, respectively. However, these effect estimates should be interpreted cautiously given their sensitivity to the high-leverage observations, particularly in the context of the relatively small sample. The nighttime pain finding additionally warrants caution because of the less consistent findings across the correlation and multivariable regression analyses. No formal statistical comparison was performed between the daytime and nighttime CST associations; therefore, these findings should not be interpreted as demonstrating a difference in the strength of the associations.

Clinical evidence regarding the relationship between CST and pain remains limited. Recently, Roglic Karan et al. reported significant positive associations between serum CST concentrations and both pain intensity and burning sensation in patients with oral lichen planus [15]. Experimental evidence has also implicated CST in pain-related pathways. In a rat model of neuropathic pain, CST administration enhanced pain-related behavior, with reduced mechanical withdrawal thresholds and thermal withdrawal latencies, and increased P2X4 receptor expression in satellite glial cells of the dorsal root ganglia [21]. These experimental findings suggest a pronociceptive effect of CST and therefore are not directly concordant with the inverse clinical association between circulating CST levels and pain severity observed in our FMS cohort. CST has also been linked to autonomic regulation, catecholamine release, and neuroimmune signaling, all of which may be relevant to chronic pain [22]. However, these findings were obtained in different clinical and experimental settings and do not directly explain the inverse association observed in our FMS cohort. The limited available evidence therefore indicates that the relationship between CST and pain warrants further investigation across different pain conditions. Taken together, the observed pattern of findings draws attention to a potential relationship between circulating CST and pain severity within FMS. Nevertheless, the clinical significance of this association remains uncertain, and its relationship to FMS itself cannot be determined from the present findings. Nighttime pain in FMS frequently coexists with sleep disturbances and non-restorative sleep, and the relationship between sleep and pain is considered bidirectional [23,24]. Altered CST levels have also been reported in sleep-related disorders, including obstructive sleep apnea [25]; however, sleep parameters were not assessed in the present study, and a sleep-related explanation cannot be inferred from our findings. Temporal neuroendocrine and autonomic variation may also warrant consideration when interpreting the different findings for daytime and nighttime pain. CST is derived from chromogranin A and is involved in sympathoadrenal regulation through modulation of catecholamine release [26], while temporal variation has been reported for chromogranin A and related neuroendocrine activity [27,28]. Nevertheless, the temporal profile of circulating CST itself remains insufficiently characterized, and CST was measured at only a single morning time point in the present study. Thus, whether temporal variation in CST or related neuroendocrine processes contributed to the observed daytime and nighttime findings cannot be determined. Overall, the nighttime pain finding should be considered preliminary and interpreted cautiously, and future studies incorporating serial CST measurements together with sleep-related and autonomic assessments may help clarify these observations.

The recently developed FM-QoLS was used in the present study to assess fibromyalgia-specific quality of life [16]. Data regarding its use in clinical research remain limited. No significant association was observed between FM-QoLS scores and serum CST levels. In contrast, the inverse association between FM-QoLS and FIQ scores remained significant after FDR correction, supporting the relationship between fibromyalgia-specific quality of life and overall disease impact. Quality of life is a multidimensional construct influenced by biological and psychosocial factors, including pain, sleep, fatigue, psychological well-being, and social functioning. The absence of an association between CST and FM-QoLS may therefore reflect the limited ability of a single circulating biomarker to capture this broader clinical construct. Taken together, these findings indicate that CST was associated with pain intensity in the present sample, whereas no statistically significant associations were observed with FIQ or FM-QoLS scores.

As the study population consisted entirely of women across a relatively broad age range (18–65 years), hormonal and reproductive factors should also be considered when interpreting the findings. Menopausal transition has been associated with differences in pain and symptom burden in FMS, and recent evidence further indicates substantial overlap between menopausal symptoms and the clinical manifestations of FMS [29,30]. Sex-related differences in circulating CST concentrations have also been reported, with higher CST levels observed in women than in men, although the extent to which these differences are attributable to sex hormones remains unclear [31]. In the present study, menopausal status, menstrual cycle phase, and circulating sex hormone levels were not systematically assessed. Although age was not associated with CST or pain severity in our cohort, menopausal and hormonal status cannot be inferred from age alone. Therefore, the potential influence of these unmeasured factors on the observed associations cannot be excluded.

Joint hypermobility was also not systematically assessed in the present study. This may be relevant because hypermobility spectrum disorders have been associated with dysautonomia and orthostatic intolerance [32], while CST is involved in autonomic and cardiovascular regulation, including blood pressure regulation [19,20]. Therefore, the potential contribution of joint hypermobility and associated autonomic or hemodynamic alterations to pain severity and the observed CST–pain associations could not be evaluated.

Several strengths of the present study should also be noted. Participants were evaluated using well-defined eligibility criteria, with several comorbidities and medications potentially relevant to CST excluded. The inclusion of a healthy control group allowed direct comparison of circulating CST levels, while the assessment of pain, disease impact, and FMS-specific quality of life provided a broader clinical characterization. In addition, correction for multiple comparisons and multivariable analyses allowed a more conservative assessment of the observed associations.

## 5. Limitations

This study has several limitations. First, its cross-sectional and single-center design precludes causal inference and limits the generalizability of the findings. The relatively small sample size, particularly the 40 patients included in the within-group analyses, also limited the number of covariates that could reasonably be included in the multivariable models. In addition, the study included only women with newly diagnosed and untreated FMS who were selected using stringent eligibility criteria. Although this approach was intended to reduce potential treatment- and comorbidity-related confounding, it resulted in a highly selected study population and may have introduced selection bias, thereby limiting the applicability of the findings to the broader FMS population. Menopausal status, menstrual cycle phase, and sex hormone levels were not systematically assessed. Sleep disturbances, fatigue, anxiety and depressive symptoms were also not specifically evaluated as study variables. Therefore, residual confounding from these unmeasured clinical and hormonal factors cannot be excluded.

Second, serum CST was measured at a single morning time point, and repeated measurements across the day were not performed; therefore, intra-individual variability and the temporal profile of circulating CST could not be evaluated. Blood pressure and heart rate were not systematically recorded at the time of blood sampling, and direct measures of autonomic function were not obtained. Moreover, chromogranin A, inflammatory cytokines, and other neuroendocrine or autonomic markers were not measured, limiting our ability to explore the biological pathways underlying the observed CST–pain associations.

Third, study samples were analyzed as single determinations; therefore, within-study analytical precision could not be estimated from replicate measurements of individual serum samples. The CVs obtained from duplicate calibration standards reflect the consistency of the calibration measurements and do not establish analytical precision for individual serum samples. No independent analytical validation of the E4996Hu assay was performed in the present study. In addition, although the assay uses catestatin-directed capture and detection antibodies, quantitative specificity/cross-reactivity data for related peptides were not available in the manufacturer documentation reviewed; therefore, the antibody-based target-recognition principle alone should not be considered quantitative evidence establishing the absence of cross-reactivity. These analytical limitations should be considered when interpreting the absolute CST concentrations and the observed associations.

## 6. Conclusions

In conclusion, serum CST levels did not differ significantly between patients with FMS and healthy controls in this sample. Within the FMS group, lower serum CST levels were associated with greater daytime pain severity, whereas no significant associations were observed with symptom duration, FIQ, or FM-QoLS scores. An inverse association with nighttime pain was also observed, although this finding should be interpreted cautiously. These findings suggest a potential relationship between circulating CST and pain severity within FMS; particularly daytime pain. However, the observed associations do not establish the underlying mechanisms linking CST to pain and should not be interpreted as evidence of the diagnostic utility of CST for FMS. The findings should therefore be considered preliminary and hypothesis-generating. Future prospective studies involving larger and more diverse populations, serial CST measurements, and more detailed characterization of pain and associated FMS symptoms are needed to confirm these observations and determine their clinical relevance. Such studies should also consider relevant potential confounders and incorporate inflammatory, neuroendocrine, and autonomic measures to further characterize the relationship between CST and pain in FMS.

## Figures and Tables

**Table 1:** Summary of previous studies investigating catestatin in rheumatic and pain-related conditions.

Disease	Study	CST Assay	Association with Disease Activity/Severity	Main Serum CST Finding
Ankylosing spondylitis	Alisik et al., 2025 [12]	ELISA; E4996Hu, BT Lab	Lower CST levels were associated with higher disease activity; CST was inversely associated with ASDAS-CRP	↓ Serum CST compared with healthy control
Rheumatoid arthritis	Simac et al., 2022 [13]	ELISA; EK-053-27CE, Phoenix Pharmaceuticals	Higher CST levels were positively associated with DAS28 and HAQ scores	↑ Serum CST compared with healthy controls
Rheumatoid arthritis	Pàmies et al., 2024 [14]	ELISA; RayBiotech	Higher CST levels were associated with RF and ACPA seropositivity	Serum CST was evaluated in RA; no healthy control group was included
Oral lichen planus	Roglic Karan et al., 2026 [15]	ELISA; EK-053-27CE, Phoenix Pharmaceuticals	Higher CST levels were associated with greater clinical severity and higher VAS pain and burning scores	↑ Serum CST compared with healthy controls

Abbreviations: CST, catestatin; ASDAS-CRP, Ankylosing Spondylitis Disease Activity Score with C-reactive protein; DAS28, Disease Activity Score in 28 joints; HAQ, Health Assessment Questionnaire; RF, rheumatoid factor; ACPA, anti-citrullinated protein antibody; RA, rheumatoid arthritis; VAS, visual analog scale. ↑ indicates higher serum CST levels compared with healthy controls; ↓ indicates lower serum CST levels compared with healthy controls.

**Table 2:** Clinical characteristics of patients with fibromyalgia syndrome.

Variable	FMS Patients (*n* = 40)
symptom duration (months)	24.00 (9.75–60.00)
VAS-day	6.68 ± 2.10
VAS-night	7.50 (5.00–9.00)
FIQ score	66.83 ± 17.28
FMQOLS score	33.16 ± 12.13

Data are presented as mean ± standard deviation (SD) for normally distributed variables and median (interquartile range, IQR) for non-normally distributed variables. VAS, Visual Analog Scale; FIQ, Fibromyalgia Impact Questionnaire; FMQOLS, Fibromyalgia Quality of Life Scale.

**Table 3:** Comparison of demographic characteristics, laboratory parameters, and serum catestatin levels between the patient and control groups.

Variable	Control (*n* = 40)	FMS (*n* = 40)	Between-Group Estimate (95% CI) †	Effect Size ‡	*p*
Age (years)	43.18 ± 9.30	44.85 ± 10.02	1.68 (−2.63 to 5.98)	0.173	0.441
BMI (kg/m^2^)	28.03 ± 4.73	28.32 ± 4.84	0.29 (−1.84 to 2.42)	0.061	0.785
Catestatin (ng/mL)	2.36 (1.99–4.74)	2.07 (1.77–3.34)	−0.285 (−0.720 to 0.060)	−0.207	0.111
CRP (mg/L)	3.33 (3.30–5.22)	3.22 (3.13–8.97)	−0.08 (−0.17 to 0.16)	−0.125	0.333
ESR (mm/h)	12.00 (6.25–17.75)	11.00 (6.00–15.75)	−1.00 (−4.00 to 2.00)	−0.094	0.467
Vitamin D (ng/mL)	16.50 (8.25–29.75)	18.00 (10.00–25.00)	1.00 (−4.00 to 5.00)	0.040	0.758

Data are presented as mean ± standard deviation (SD) for normally distributed variables and median (interquartile range, IQR) for non-normally distributed variables. Between-group comparisons were performed using the independent samples *t*-test for normally distributed variables and the Mann–Whitney U test for non-normally distributed variables. **†** Between-group estimates were calculated as FMS minus control and are presented as mean differences for normally distributed variables and Hodges–Lehmann location-shift estimates for non-normally distributed variables, with 95% confidence intervals (CIs). **‡** Effect sizes are expressed as Cohen’s d for normally distributed variables and rank-biserial correlation (r_rb) for non-normally distributed variables. BMI, body mass index; CRP, C-reactive protein; CST, catestatin; ESR, erythrocyte sedimentation rate.

**Table 4:** Correlations between serum catestatin levels and disease-related clinical parameters in patients with fibromyalgia syndrome.

Variable 1	Variable 2	Spearman’s ρ	Unadjusted *p*	FDR-Adjusted q
CST	Symptom duration	0.030	0.856	0.917
CST	VAS-day	−0.510	0.001	**0.008**
CST	VAS-night	−0.364	0.021	0.053
CST	FIQ	−0.120	0.459	0.765
CST	FM-QoLS	0.048	0.767	0.917
symptom duration	VAS-day	−0.067	0.679	0.917
symptom duration	VAS-night	0.259	0.107	0.229
symptom duration	FIQ	−0.010	0.949	0.949
Symptom duration	FM-QoLS	−0.041	0.803	0.917
VAS-day	VAS-night	0.721	<0.001	**<0.008**
VAS-day	FIQ	0.400	0.011	**0.033**
VAS-day	FM-QoLS	−0.106	0.514	0.771
VAS-night	FIQ	0.480	0.002	**0.008**
VAS-night	FM-QoLS	−0.236	0.142	0.266
FIQ	FM-QoLS	−0.485	0.002	**0.008**

*p* values are two-sided and are presented as unadjusted values. The Benjamini–Hochberg false discovery rate (FDR) procedure was applied to the 15 unique pairwise correlations, and the corresponding FDR-adjusted *p* values are reported as q values. Statistical significance after correction was defined as q < 0.05. Bold values indicate correlations that remained statistically significant after FDR correction. VAS, Visual Analog Scale; FIQ, Fibromyalgia Impact Questionnaire; FM-QoLS, Fibromyalgia Quality of Life Scale.

**Table 5:** Multiple linear regression analyses of factors associated with daytime and nighttime pain intensity in patients with fibromyalgia syndrome.

Outcome/Predictor	B	SE	Standardized β	95% CI for B	*p*
**VAS-day**					
Catestatin	−0.175	0.045	−0.541	−0.267 to −0.083	**<0.001**
Symptom duration (months)	0.005	0.013	0.054	−0.022 to 0.032	0.732
Age (years)	−0.023	0.034	−0.112	−0.093 to 0.046	0.499
BMI (kg/m^2^)	0.061	0.068	0.140	−0.076 to 0.198	0.373
**VAS-night**					
Catestatin	−0.222	0.056	−0.530	−0.336 to −0.107	**<0.001**
Symptom duration (months)	0.035	0.017	0.318	0.001 to 0.069	**0.041**
Age (years)	−0.011	0.043	−0.040	−0.098 to 0.076	0.800
BMI (kg/m^2^)	−0.010	0.084	−0.018	−0.181 to 0.161	0.904

Separate multiple linear regression models were constructed for daytime and nighttime VAS scores. Serum catestatin, age, BMI, and symptom duration were entered simultaneously into each model. For the VAS-day model, R^2^ = 0.314, adjusted R^2^ = 0.236, F(4,35) = 4.007, *p* = 0.009. For the VAS-night model, R^2^ = 0.368, adjusted R^2^ = 0.296, F(4,35) = 5.096, *p* = 0.002. B, unstandardized regression coefficient; SE, standard error; β, standardized regression coefficient; CI, confidence interval; BMI, body mass index; VAS, visual analog scale. Bold values indicate statistically significant results (*p* < 0.05).

## Data Availability

The data that support the findings of this study are available from the corresponding author upon reasonable request.

## Supplementary Materials

The following supporting information can be downloaded at: https://www.mdpi.com/article/10.3390/jcm15187332/s1, Table S1: Comparison of additional routine laboratory parameters between the patient and control groups; Table S2: Bootstrap confidence intervals for correlations between serum catestatin and clinical parameters in patients with fibromyalgia syndrome.
