# Supplementary material for: Serum Catestatin Levels and Their Association with Pain Severity in Fibromyalgia Syndrome: A Cross-Sectional Case–Control Study
Source: J Clin Med. 2026 Sep 21;15(18):7332. doi: 10.3390/jcm15187332 (PMC13607623; doi:10.3390/jcm15187332)
Supplement: Supplementary file 1 [file jcm-15-07332-s001.zip › jcm-4534881-supplementary.pdf]

Supplementary Table S1. Comparison of additional routine laboratory parameters between the patient and control groups

Variable	Control (n=40)	FMS (n=40)	Between-group estimate (95% CI)†	Effect size‡	p
Hemoglobin (g/dL)	13.00 (11.85–13.60)	13.10 (12.33–13.98)	0.20 (−0.40 to 0.80)	0.082	0.528
WBC (×10³/μL)	7.64 (6.17–9.10)	7.48 (6.45–8.89)	−0.095 (−1.04 to 0.89)	−0.023	0.862
Platelet (×10³/μL)	290.45 ± 59.11	303.63 ± 74.13	13.18 (−16.67 to 43.02)	0.197	0.382
Total cholesterol (mg/dL)	198.53 ± 33.54	198.93 ± 41.42	0.40 (−16.38 to 17.18)	0.011	0.962
LDL-C (mg/dL)	120.18 ± 29.83	120.08 ± 33.88	−0.10 (−14.31 to 14.11)	−0.003	0.989
HDL-C (mg/dL)	56.53 ± 11.64	53.53 ± 12.31	−3.00 (−8.33 to 2.33)	−0.250	0.266
Triglycerides (mg/dL)	127.00 (93.50–175.50)	139.50 (94.75–193.75)	12.00 (−16.00 to 42.00)	0.108	0.408
ALT (U/L)	16.00 (13.00–22.25)	13.50 (10.25–18.00)	−2.00 (−5.00 to 0.00)	−0.244	0.059
AST (U/L)	17.00 (14.00–19.75)	15.00 (14.00–18.75)	−1.00 (−3.00 to 1.00)	−0.138	0.285
TSH (mIU/L)	1.50 (1.10–2.24)	2.02 (1.17–3.29)	0.415 (0.000 to 0.960)	0.251	0.054
ALP (U/L)	68.50 (56.25–87.00)	76.00 (69.00–101.75)	11.00 (−1.00 to 19.00)	0.242	0.062
Creatinine (mg/dL)	0.67 ± 0.10	0.71 ± 0.10	0.039 (−0.006 to 0.083)	0.388	0.087

Data are presented as mean ± standard deviation (SD) for normally distributed variables and median (interquartile range, IQR) for non-normally distributed variables. Between-group comparisons were performed using the independent samples t-test for normally distributed variables and the Mann–Whitney U test for non-normally distributed variables.

†Between-group estimates were calculated as FMS minus control and are presented as mean differences for normally distributed variables and Hodges–Lehmann location-shift estimates for non-normally distributed variables, with 95% confidence intervals (CIs).

‡Effect sizes are expressed as Cohen's d for normally distributed variables and rank-biserial correlation (r_rb) for non-normally distributed variables.

WBC, white blood cell count; LDL-C, low-density lipoprotein cholesterol; HDL-C, high-density lipoprotein cholesterol; ALT, alanine aminotransferase; AST, aspartate aminotransferase; TSH, thyroid-stimulating hormone; ALP, alkaline phosphatase.

Supplementary Table S2. Bootstrap confidence intervals for correlations between serum catestatin and clinical parameters in patients with fibromyalgia syndrome

**Clinical parameter**	**Spearman's ρ**	**95% BCa bootstrap CI**	**Unadjusted *p***	**FDR-adjusted *q***
VAS-day	−0.510	−0.746 to −0.164	**0.001**	**0.008**
VAS-night	−0.364	−0.642 to 0.000	0.021	0.053
FIQ	−0.120	−0.410 to 0.192	0.459	0.765
FM-QoLS	0.048	−0.259 to 0.365	0.767	0.917

CST, catestatin; CI, confidence interval; BCa, bias-corrected and accelerated; VAS, Visual Analog Scale; FIQ, Fibromyalgia Impact Questionnaire; FM-QoLS, Fibromyalgia Quality of Life Scale. The 95% confidence intervals for Spearman's ρ were estimated using BCa bootstrap resampling with 5,000 paired resamples. Unadjusted *p*-values are two-sided. FDR-adjusted *q*-values were obtained using the Benjamini–Hochberg procedure applied to the 15 unique pairwise correlations presented in Table 4. Statistical significance after FDR correction was defined as $q < 0.05$.
